# Recent Progress in Research on the Pathogenesis of Pulmonary Thromboembolism: An Old Story with New Perspectives

**DOI:** 10.1155/2017/6516791

**Published:** 2017-04-06

**Authors:** Chao Yan, Xiaohua Wang, Hua Su, Kejing Ying

**Affiliations:** Department of Respiratory Medicine, Sir Run Run Shaw Hospital, School of Medicine, Zhejiang University, Hangzhou 310016, China

## Abstract

Pulmonary thromboembolism (PTE) is part of a larger clinicopathological entity, venous thromboembolism. It is also a complex, multifactorial disorder divided into four major disease processes including venous thrombosis, thrombus in transit, acute pulmonary embolism, and pulmonary circulation reconstruction. Even when treated, some patients develop chronic thromboembolic pulmonary hypertension. PTE is also a common fatal type of pulmonary vascular disease worldwide, but earlier studies primarily focused on the pathological changes in the blood component of the disease. With contemporary advances in molecular and cellular biology, people are becoming increasingly aware of coagulation pathways, the function of vascular smooth muscle cells, microparticles, and the inflammatory pathways that play key roles in PTE. Combined hypoxia and immune research has revealed that PTE should be regarded as a class of complex diseases caused by multiple factors involving the vascular microenvironment and vascular cell dysfunction.

## 1. Introduction

Venous thromboembolism (VTE), a combination of deep venous thrombosis (DVT) and pulmonary embolism (PE), is a major cause of morbidity and death in patients worldwide. PE is a common and potentially fatal disease that is caused by a perfusion defect due to an embolus blocking blood flow in the lungs [1]. VTE comprises all types of venous thrombosis in the various compartments, whether superficial or in the deep veins. PTE is also regarded as an acute complication of DVT [2]. The incidence of VTE is 1 : 1000 per year, and that of PE is approximately 50 in 100,000 per year in Europe [3, 4]. Similarly, in the USA it affects an estimated number of 900,000 people each year, resulting in large numbers of hospitalizations and approximately 300,000 deaths [5].

Clinically, based on the classification of patients with acute PE based on early mortality risk, patients with high-risk PE (presenting with shock or hypotension) need to receive reperfusion therapy with thrombolysis, and low-risk (pulmonary embolism severity index [PESI] classes I-II or simplified pulmonary embolism severity index [sPESI] = 0) and intermediate-risk (PESI classes III-IV or sPESI ≥ 1) PEs are usually treated with anticoagulation and thrombolysis therapies to reconstruct the normal pulmonary hemodynamics [6]. The process of thrombolysis depends on two methods of clearing blood clots: thrombus dissolution by the endogenous fibrinolytic system and thrombus recanalization by the vascular microenvironment [7, 8]. In addition to traditional anticoagulants (e.g., heparin, warfarin) [9], novel anticoagulant drugs (e.g., dabigatran, rivaroxaban) and new medical materials have also played an important role in the prevention and treatment of PTE [10, 11]. However, confusion still exists for certain patients. Even after formal treatment, such as continuous anticoagulant therapy for 3–6 months, normal pulmonary circulatory function cannot be reconstructed after PTE. These patients often end up with chronic thromboembolic pulmonary hypertension (CTEPH) [12].

In 1856, Virchow concluded that vessel wall injury, blood stasis, and hypercoagulability are the three synergic abnormalities that cause VTE [13]. The critical event in venous thrombosis is thrombin generation from the coagulation pathways, initialized by activated tissue factor (TF) [14]. In contrast, thrombus elimination is the result of a long-term interaction between the fibrinolysis system and the vascular microenvironment [15, 16]. Many discoveries from recent studies have shown that the activation and signal transduction of protease activated receptors (PARs), pulmonary artery smooth muscle cells (PASMCs) responses to hypoxia, cell-cell interactions mediated by extracellular vesicles (EVs), such as microparticles (MPs), and inflammation are the diverse mechanisms associated with the occurrence and development of PTE. This review discusses these potential mechanisms and aims to elucidate the detailed pathophysiology of PTE.

## 2. PARs: Activation and Venous Thrombosis

The initial event of PTE is venous thrombosis [14]. As a result, PE is considered an acute complication of DVT, and postthrombotic syndrome (PTS) is a long-term complication of DVT [17]. Therefore, a close relationship has been observed between PTE and coagulatory function. PARs serve as a significant driving force in the coagulation cascade and signal transduction pathways [18–20], and they may play an extremely important role in the process of PTE. PARs are seven-transmembrane G-protein-coupled receptors and include four family members, named PAR1–PAR4 [21]. PAR1, PAR3, and PAR4 are mainly cleaved and activated by thrombin, which acts as a key serine protease in the coagulation cascade [22–24]. However, PAR2 is a receptor for mast cell tryptase, coagulation factors VIIa and Xa, and trypsin [25, 26]. This family is widely distributed throughout various organs of the body, such as the cardiovascular system, respiratory system, nervous system, and renal system [27]. Particularly, in the circulation system, it can promote the activation of platelets [28] or endothelial cells [29] and TF regulation in the pathological process [30]. PAR4-deficient mice are protected against thromboplastin-induced PE [31]. However, it is important to note that there are species differences in the expression of PARs in platelets. For example, rat and mice platelets lack PAR1 [32, 33].

The activation of PARs requires proteolytic cleavage at the N-terminal extracellular domain, which generates a new N-terminal domain that functions as a tethered ligand by binding intramolecularly to the receptor, triggering transmembrane signaling [18]. PAR-agonist peptides (PAR-APs) are structurally similar to the tethered ligands and can activate PARs independently of protease activity and receptor cleavage [26]. Activated PARs initiate cell signaling via the recruitment of heterotrimeric G proteins, including Gi, G12/13, and Gq [21]. Subsequently, downstream cellular responses lead to nociception, inflammation, cell migration, and proliferation, which often occur in pulmonary diseases [34].

PARs are expressed on both endothelial cells (ECs) and vascular smooth muscle cells (VSMCs). ECs mainly express PAR1, and other types of PARs are also present in very low amounts [35]. To represent typical vascular endothelial cells (VECs), human umbilical vein endothelial cells (HUVECs) and pulmonary artery endothelial cells (PAECs) are used to study the relationship between ECs and coagulation function [36, 37]. It has been demonstrated that TF-FVIIa-Xa complexes can transmit signals by PAR1 and TF-FVIIa complexes directly have a function that activates PAR2 [38]. These significant events occur during the process of clot formation and increased pulmonary vascular resistance in PTE.

However, studies suggest that the activation of TF and PARs is far more complex than once thought [39]. After specific peptide binding and activation, PAR1 and PAR2, which are expressed in HUVECs, and TF, which is expressed in ECs, can be induced by the redox-sensitive signaling pathway. The key signaling molecules of the common pathway are reactive oxygen species (ROS) generated by the mitochondrial electron transport chain and simultaneously activated ERK1/2 and MAPKp38. Mitochondrial complex III is mostly involved in the generation of ROS induced by PAR1 and PAR2. Researchers have also discovered that thrombin and PAR2-AP have a similar pharmacological activity to induce the production of TF mediated by mitochondrial complexes I and III [39, 40]. This finding contradicts the previous theory that the TF-VIIa complex activates PARs signal transduction independently [38]. Low concentrations of thrombin, which accumulates in the valve pocket, and local prolonged hypoxia caused by blood stasis are the two major characteristics of the initiation process of venous thrombosis [41, 42]. In addition, the expression of TF at the embolism site is not significantly increased [43]. A reasonable assumption is that if TF is activated strongly and immediately on VECs through mROS, which are induced by thrombin or hypoxia in acute PE, then it may play a central role in the triggering and spread of thrombi. This mechanism can explain the strengthened process of thrombosis in the acute phase of PTE. It also demonstrates that activation and regulation of TF and PARs are bidirectional.

Previous studies have shown that venous thrombi are mainly composed of fibrin and red blood cells [44, 45], and the role and function of platelets in VTE are rarely implicated. However, many recent studies show that platelets are a very important factor in thromboembolic disease. It was noted that venous thrombi contain platelets, and platelet activation is associated with thrombus initiation and propagation [46, 47]. PARs are important in multiple regulation pathways of platelet activation and directly or indirectly affect the progression of PTE. It is known that PAR1 can mediate the activation of human platelets by thrombin at low concentrations, but PAR4 plays a similar role at high thrombin concentrations if PAR1 is absent [18]. Recent research has shown that Prohibitin l (PHB1), expressed on the platelet membrane, has the function of microcontrol action on the PAR1 signaling pathway [48]. Experiments have confirmed that PHB1 is a type of membrane protein from human platelets that participates in the pathological activation of PAR1 and is induced by low concentrations of thrombin in PAR1-mediated platelet aggregation. Once PAR1 in HUVECs is activated, PHB1 also has an impact on the degradation and internalization of PAR1 [49].

## 3. Functional Proteins in PASMCs: A Link between Pulmonary Vasoconstriction and Hypoxia

The pulmonary vascular wall consists of three layers: the adventitia, media, and intima [50]. PASMCs located in the media are highly specialized cells. Differentiated SMCs (also called the “contractile phenotype”) differ from other types of blood vessel cells, and their proliferation and synthesis abilities are inhibited [15, 51]. Under the effect of oxygen concentrations, PASMCs maintain vascular tension through the regulation of contraction and relaxation [52, 53]. They can express proteins associated with contraction function [54] and ion channels that participate in the process of pulmonary vasoconstriction [55, 56].

Arterial hypoxemia, caused by the mismatching of ventilation and perfusion, is due to a thrombus obstructing the pulmonary artery or its branches in acute PE [57]. Hypoxic pulmonary vasoconstriction (HPV) can also elevate the pulmonary artery pressure (PAP) by increasing the pulmonary vascular resistance [58]. The exact mechanism has not been fully clarified, but PASMCs play an important role in HPV. Previous studies have suggested that oxygen concentrations can be “sensed” by mitochondria [40] or NADPH oxidases [59] from PASMCs related to the alteration of ROS. There is a debate in the literature regarding whether ROS levels increase or decrease during hypoxia [60–62]. However, it is known that ROS has the function of regulating the K^+^ and Ca^2+^ channels in PASMCs [63, 64]. Redox-sensitive K^+^ channels were found to be inhibited under hypoxia, and membrane potential changes and voltage-gated L-type Ca^2+^ channels were activated. Elevated intracellular Ca^2+^ concentrations from the internal release of calcium, or the influx of extracellular calcium, produce signals that trigger PASMCs contraction by activating actin and myosin.

Presently, the theory regarding the precise mechanism of HPV remains unclear. For example, the functions of actin-associated proteins in PTE are largely unknown. We have shown that hypoxia can regulate the production of two actin-associated proteins through the hypoxia inducible factor (HIF) pathway in human or rat PASMCs. HIF-1*α* can induce the expression of the capping protein CapG, and HIF-2*α* can induce the expression of transgelin (SM22) [65, 66]. CapG is an actin regulatory protein that can modulate actin length by binding and capping the end of actin filaments in a Ca^2+^- and polyphosphoinositide-dependent manner [67]. Transgelin can participate in the organization of actin distribution by interacting with actin and plays an important role in the regulation of PASMCs contraction in a Ca^2+^-independent manner [68, 69]. The upregulation of these two proteins contributes to the increased motility and contraction of PASMCs under acute or sustained exposure to low-oxygen environments. These observations indicate that the two oxygen-sensing pathways that are dependent on ROS or HIF may both play a significant role in pulmonary vasoconstriction after PE.

Acute hypoxia in PTE causes clinical manifestations, including vascular contraction, alveolar hypoxia, and the increased blood coagulation activity [42, 70]. However, unlike acute hypoxia, sustained hypoxia is more common in a variety of pulmonary vascular diseases [71]. The upregulation of transgelin and CapG enhances the migratory ability of PASMCs during the process of vascular remodeling caused by chronic hypoxia [66, 72]. In future studies, it would be interesting to focus on the differences in the mechanism between CTEPH associated with PTE and hypoxic pulmonary hypertension (HPH), due to lung disease and/or hypoxia. Hypoxia also overcomes the balance of ACE/ACE2 expression levels by HIF-1*α* [73] and plays key roles in promoting the contraction and proliferation of PASMCs by inhibiting the ACE2-Ang(1–7)-Mas axis. The administration of recombinant ACE2 suppresses the pulmonary vasoconstriction response to acute hypoxia that occurs in pulmonary hypertension (PH) related to high altitude [74], which is similar to the symptoms of acute PE. Moreover, the functions of the contraction, secretion, growth, and migration of PASMCs greatly affect thrombus dissolution and recanalization, which are closely associated with the prognosis of an individual patient after acute PE.

## 4. Microparticles: A Potential Biomarker for PTE

EVs are nanosized, membrane-limited vesicles released from cells that participate in cell-cell communication [75]. Several types of cells are capable of releasing EVs, which can transport DNA, RNA, lipids, and proteins [76] by shedding vesicles from their plasma membrane. Due to the release of EVs from cells throughout the body, they can also be detected in diverse body fluids [75] and in cell culture supernatants [77]. Cells produce different subtypes of EVs that vary in size, including microvesicles (MVs)/microparticles (MPs), exosomes, oncosomes, and apoptotic bodies [78]. MVs and exosomes are generated by normal and cancer cells [76]. The mechanism of intercellular information transmission by these two major types of EVs has also been extensively studied. MVs that contain cytoplasmic cargos are 0.1–1 *μ*m in diameter and come directly from the plasma membrane [78, 79]. MPs and exosomes can be released from VECs and VSMCs induced by hypoxia [80], shear stress [81], or cytokines [82] during the disease process. Therefore, they are regarded as potential biomarkers for the diagnosis or prognosis of PTE and provide a new treatment strategy in addition to anticoagulation and thrombolysis [83].

MPs seem to be associated with a hypercoagulable state, which predisposes a person to thrombosis but does not determine its occurrence. The levels of circulating tissue factor bearing MP (TF^+^MPs) [84] or phosphatidylserine (PS^+^) and lactadherin^+^ MPs [85] are higher in patients with a hypercoagulable status than in control subjects. Elevated circulating MP-TF activity is associated with thrombosis and worsened survival in patients with pancreaticobiliary cancers (PBCs) [86]. Increased levels of glial-derived and/or TF^+^MPs are also noted in glioblastoma multiforme (GBM) patients both before and even more so after the neoplasm is treated, suggesting a contribution of TF^+^/GFAP-MPs to the risk of VTE [87]. Watts and colleagues [88] demonstrated that MP protein composition changes and the proteins involved in clot formation increase during PE compared with controls. The process of acute PE in a rat model induced by injection with polystyrene microspheres also showed that the proteins inside MPs have prothrombotic characteristics related to fibronectin, fibrinogen, and von Willebrand factor. However, in another study, Garcia Rodriguez and colleagues [89] demonstrated that plasma MP-TF activity in patients presenting with clinically suspected PE was not associated with confirmed PE. In both groups of patients presenting with symptoms of PE, the median MP-TF activities were significantly higher than those in the healthy controls, especially in patients with the presence of active cancer or cardiovascular disease. These findings suggest that the high MP-TF activity levels found in cancer patients with acute VTE originate from aggressive tumour cells rather than an acute thromboembolic event.

Valuable biomarker candidates must possess specificity, sensitivity, timeliness, and a biological gradient. Associations between elevated MPs levels and an increased risk of VTE have been found in patients with underlying diseases such as high-grade glioma [90] or in patients undergoing in vitro fertilization [91] to treat infertility. However, confusion and contradictions are still present in clinical studies [92, 93]. Further research should focus on the following problems:

- The limitations of current assays in measuring MPs may be divided into sizing, probing, and counting [94]. The resultant difference between flow cytometry and other methods such as enzyme-linked immunosorbent assays is a widespread problem because a standardized conversion between the two methods is difficult and lacking.
- Some studies have shown that PS- or TF-dependent procoagulant activity is consistent with a hypercoagulable status and the occurrence of VTE [85, 95]. Further studies are necessary to identify the diagnostic value and clinical significance of different procoagulant markers from MPs.
- MPs are derived from various cells, including blood cells, ECs, SMCs, and malignant cells [96]. Mesenchymal stem cells, which are applied to biotherapy, can also release MVs [97], and PTE is one of the most serious complications during the treatment procedures [98]. Thus, distinguishing the source of MPs is one of the core problems associated with using MPs as a biomarker to diagnose or assess the prognosis of PTE.
- In the process of collecting and detecting blood samples, researchers often overlook the fact that different treatment methods or drug applications involving anticoagulants have potential effects on the production or function of TF^+^MPs [99]. Antitumor therapies, such as chemotherapy, may also increase the level and/or activity of TF^+^MP [100, 101].

## 5. Inflammation: A Close Association with the Occurrence of PTE

Under the condition of multiple stimuli such as hypoxia or trauma, inflammation is a trigger for pulmonary endothelial dysfunction and platelet activation [102, 103]. Evidence has implicated the presence of inflammation with acute PE, including the observation that the white blood cell (WBC) count and neutrophil-to-lymphocyte ratio (NLR) are associated with short-term outcomes in PE patients. Venetz et al. [104] demonstrated that patients with an elevated WBC count (>9.8 × 10^9^/L) have a significantly higher 30-day mortality than patients who do not, after adjustment for thrombolytic therapy and for patient- and hospital-related confounders. Previous studies [105] have shown that an elevated WBC count may be a marker for hypercoagulability and that the WBC count correlates with levels of fibrinogen, factor VII, and factor VIII. A retrospective cohort study [106], including 667 PE patients, further showed that leukocytosis and the systemic inflammatory response are prognostic factors for 30-day mortality after PE. Other researchers have also observed that the platelet-to-lymphocyte ratio (PLR) is an independent predictor of mortality in acute PE patients, which is significantly correlated with PESI scores [107]. Moreover, the NLR is considered a better independent predictor of in-hospital mortality and may be used for clinical risk classification because of its reliability in the distinction between massive and submassive embolism [108].

Furthermore, the key role of neutrophils in PTE has been confirmed by a series of experimental studies. Cytokine-induced neutrophil chemoattractant-1 (CINC-1) expression increased 18- and 24-fold at 6 and 18 h after PE, respectively, and an influx of neutrophils was observed, with a significant upregulation 18 h after PE [109]. Neutrophilic inflammation is also observed in the lungs in an acute PE model in which bronchoalveolar lavage-associated neutrophils showed an almost 6-fold increase in rats with severe PTE compared with controls or rats with moderate PTE [110]. These results show that neutrophils contribute to right ventricular dysfunction and lung damage in rat PE. However, there was a remarkable difference between acute PH caused by PTE and chronic PH caused by HPH or CTEPH [111]. Some scholars believe that inflammation is an important pathological process of right ventricular damage after PE and can indirectly lead to a poor clinical outcome [112].

An increasing number of studies have also found that patients with chronic inflammatory disorders of the airway, such as asthma and chronic obstructive pulmonary disease (COPD), are at high risk of PTE. In a population-based, case-control retrospective study [113] that included 909,638 individuals (429,962 males and 479,676 females) aged over 14 years and 55,500 (6.1%) individuals suffering from asthma, the prevalence of PE was found to be substantially higher among asthmatic patients than in the nonasthmatic general population (0.26% versus 0.17%). A retrospective study [114] that included 648 patients with asthma (283 with severe and 365 patients with mild-to-moderate asthma) showed an almost 9-fold higher risk of PE in patients with severe asthma and a 3.5-fold higher risk of PE in patients with mild-to-moderate asthma than in the general population. Oral corticosteroid use was also found to be an independent risk factor for PE. In a large cohort study [115] that included 31,356 asthmatic patients and 125,157 nonasthmatic controls, the overall incidence rate of PE showed a 3.30-fold increase in the asthmatic patients compared with the nonasthmatic cohort (10.2 versus 3.09 per 100,000 person-years). One hypothesis is that asthma may reduce the process of clot retraction, which makes the thrombus stronger and more resistant to shear stresses and fibrinolysis. Recent studies have confirmed that asthma is associated with a significant inhibition of clot retraction [116]. The potential mechanism is that reactive nitrogen species produced in the lungs of asthmatic patients may reduce platelet contractility through the diminution of platelet energy production.

Several studies have also shown an increased prevalence of PE in COPD patients. COPD is associated with an increased risk of VTE, and PE presentation is more significantly associated with COPD patients than non-COPD patients (OR 1.64, 95% CI 1.49–1.80) [117]. Specifically, COPD is associated with an increased risk of mortality (10.8% versus 7.6%), minor bleeding (4.5% versus 2.3%), and first VTE recurrences as PE (1.5% versus 1.1%) during the 3-month follow-up. Similar to asthma, COPD patients present more frequently with PE than with DVT [114, 117]. A retrospective population-based cohort study [118] using data retrieved from Taiwan's National Health Insurance Research Database (2000 to 2008), including 355,878 COPD patients and 355,878 comparison patients, shows that the prevalence of PE in COPD patients is 3.45-fold higher than that in non-COPD patients and increases with age. Severely exacerbated COPD patients, especially those with immobility/obesity [119] or those requiring ICU admission [120], have an increased risk of PE.

The complex relationship between PTE and respiratory allergy/inflammatory diseases is partly attributed to various types of inflammatory cytokines and immune cells. Proinflammatory activity is one of the predominant features in interleukin family members [121], but some members have an anti-inflammatory function [122]. For example, M1- or M2- type macrophages play different roles in tissue damage and inflammatory responses [123]. More importantly, neutrophils are simultaneously involved in thrombosis and thrombolysis, which are two key processes in PTE. Neutrophil extracellular traps (NETs) are considered to be one of the basic structures of thrombosis [124], and the dissolution of NETs can also affect thrombus dissolution and recanalization [125]. Anti-inflammatory treatment for PTE is not as simple as anticoagulation therapy, but it will be a focus of future studies because of its potential implications in PTE.

## 6. Summary and Perspective

PTE is more closely associated with coagulation function than lung cancer, pneumonia, and other common respiratory diseases. Virchow's Triad preliminarily revealed the internal relationships between coagulant function abnormality and pathological changes in the vessel wall, based on venous thrombosis, which is the initial event of pulmonary thromboembolism. Focusing on a molecular and cellular view, contemporary research reveals that the function of coagulation involving vascular endothelium in PTE is affected by hypoxia or inflammation, and developing an efficient test for the diagnosis and prognosis of PTE depends on the knowledge of the balance between prothrombotic and antithrombotic factors in the lung.

**Figure 1:**
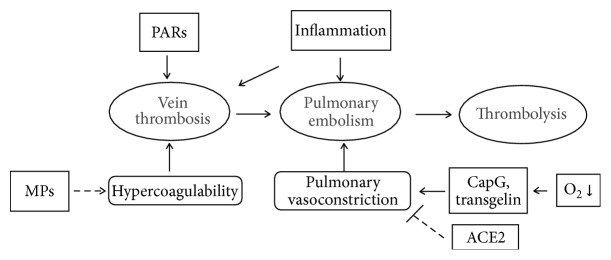
Protease activated receptors (PARs), pulmonary artery smooth muscle cells (PASMCs), microparticles (MPs), and inflammation are associated with pulmonary thromboembolism (PTE). Biomedical research has shown that PARs modulate thrombosis by activating ECs, and functional proteins (e.g., CapG, transgelin, and ACE2) in PASMCs have an important function in pulmonary vasoconstriction. Clinical research links MPs and hypercoagulability to venous thromboembolism. Epidemiological studies have shown that inflammation is a risk factor for PTE.

The aim of contemporary research is to understand the pathogenesis of PTE (Figure 1). Various laboratory studies have demonstrated the importance of PAR activation, hypoxia signaling pathways, and the generation of MPs. However, the inflammatory response is a link involving the above three events in PTE and may have an overall influence on signal transduction in the progression of the disease, depending on ECs or platelets [8, 126, 127]. Compared with traditional anticoagulation and thrombolysis therapy based on the theory of thrombosis, the activation and regulation of inflammatory pathways are complex. For example, inflammasomes are activated in diseases associated with sterile inflammation [128] and can sometimes be initiated by peptide secretions of the host at earlier stages of infection or trauma, to protect the host against microbial infection [129]. Some inflammatory factors also have a function as a two-way switch, and specific types of leukocytes, such as neutrophils, participate in the two important processes of thrombosis and thrombolysis in PTE. This finding suggests that immunization therapy for PTE is destined to be useful and that a purely anti-inflammatory treatment cannot be used, periodically, to suppress the inflammatory process. Inflammation and metabolism also have a very complex link. Extracellular ATP has a proinflammatory role in the process of infection, and this effect is produced by the activation of inflammasomes [130]. Additionally, hypoxia induces the caspase-1-mediated activation of the NALP3 inflammasome in chronic HPH [131].

Numerous clinical studies involving PTE have summarized the spectrum of risk factors associated with the occurrence and development of disease [132], and the prevention and treatment of PTE have a high rate of success based on current risk stratification. Similarly, the complex cellular and molecular mechanisms must also be explained through basic research which may generate new therapeutic strategies. Due to the improvements in animal models [133], research developments in the field of PH are more comprehensive and have a more specific theoretical depth than do those in the field of PTE. It is worth learning from the experiences gained from studies in PH. In addition to anticoagulant and thrombolytic therapy, new targeted drugs and treatments must be perfected based on the progress of PTE animal models and research methods, as well as on integrating, the involvement of anticoagulants, procoagulants, fibrinolytics, and the immune system, rather than focusing solely on the thrombus.
